# Correlation Between ABO Blood Grouping and Erythrocyte Sedimentation Rate (ESR) in Periodontal Diseases

**DOI:** 10.7759/cureus.65815

**Published:** 2024-07-31

**Authors:** Khushboo J Durge, Ruchita T Patil, Ranu R Oza, Prasad V Dhadse, Shrishti S Salian

**Affiliations:** 1 Department of Periodontology, Sharad Pawar Dental College and Hospital, Datta Meghe Institute of Higher Education & Research, Wardha, IND

**Keywords:** infection, gingiva, blood group, blood investigations, periodontitis

## Abstract

This study investigates the relationship between erythrocyte sedimentation rate (ESR) and ABO blood grouping in the context of periodontal disorders. Periodontal diseases, chronic inflammatory conditions that damage the tissues supporting teeth, can lead to tooth loss if left untreated. ESR, a common hematological test indicating systemic inflammation, has been linked to the severity of periodontal disease. ABO blood grouping, a hereditary trait, is linked to a higher risk of various oral and systemic diseases. By synthesizing existing research, this study explores the potential of the connection between blood type ABO and ESR levels in individuals with periodontal disorders, examining studies that assess the distribution of ABO blood types and corresponding ESR values among periodontal patients.

## Introduction and background

Periodontal diseases, including periodontitis (PD) and gingivitis, are rooted in chronic immune-inflammatory responses. These diseases activate plasma cells, leucocytes, neutrophils, T-lymphocytes, and chemical mediators such as chemokines, cytokines, and C-reactive proteins. Along with dental caries, they are the leading causes of tooth loss and are common worldwide [1]. Risk factors include dental health, age, gender, immunity, smoking, medication use, drug abuse, and socioeconomic status. Despite variations in periodontal disease types, all involve complex host-microbe interactions, balancing tissue destruction and homeostasis during disease progression [2].

The system of ABO blood groups, the most significant for transfusions and organ transplants, also influences infection response and other physiological processes. The four blood types-O, A, B, and AB-differ in their antigen presence and antibody production. Blood group O erythrocytes lack true antigens but have antibodies against A and B antigens. Blood groups A and B carry their respective antigens and produce antibodies against the other antigens [3]. Blood group AB erythrocytes, containing both A and B antigens, do not produce antibodies against other blood types. ABO blood types are linked to various diseases, including cancer, infections, and digestive disorders. Cytokines like interleukin-6, TNF-alpha, and high-sensitivity C-reactive proteins are elevated in PD, potentially inhibiting erythropoiesis and affecting erythrocyte sedimentation [4].

Roberts discussed the genetic basis for family susceptibility and the correlation between ABO blood type and chronic illness vulnerability [4]. Numerous studies in India and the West have examined the connection between ABO blood type and systemic illnesses, finding strong correlations with oral cancer, salivary gland tumors, and dental caries [5]. Different blood group phenotypes show varying susceptibilities to diseases [6]. Blood group A is linked to higher risks of ovarian and pancreatic cancers, gallstones, and colitis [7]. Blood types A and O may have a higher likelihood of developing diabetes mellitus [8]. Ischemic heart disease is connected to the Lewis and ABO blood groups, while stroke is linked to blood group ABO and obesity to blood group Lewis [9]. PD shares genetic components with these conditions [10].

## Review

Association of the ABO blood group with the incidence of chronic PD

Chronic PD is a common, gradually progressing condition affecting adults and the elderly globally. Studies reveal varying prevalence rates of PD among different ABO blood groups [11]. Koregol et al. found that blood group A had a notably higher percentage of periodontal disorders, while blood group AB had the lowest [12]. Rh-positive individuals were more common in all groups [12]. Demir et al. observed a high percentage of chronic PD and gingivitis in blood type O patients, with a correlation between borderline gingivitis and the Rh (+) factor [7]. Vivek et al. found that blood group O and Rh-positive individuals were more likely to develop PD [13]. Mostafa et al. noted that blood type O patients had a higher chance of developing chronic PD, with a higher distribution of Rh-positive individuals [14]. Gautam et al. concluded that blood group B had a higher prevalence of PD and gingivitis, with Rhesus-positive groups showing considerably greater rates of these conditions [15].

Association of the ABO blood group with the incidence of aggressive PD

Aggressive PD is a destructive disease characterized by the following: the involvement of multiple teeth with a distinctive pattern of periodontal tissue loss; a high rate of disease progression; an early age of onset; and the absence of systemic diseases [16]. Aggressive PD involves an inflammatory response leading to alveolar bone loss, attachment loss, and connective tissue damage. Kaslick et al. found that blood group O patients were significantly less common than blood group B patients [17]. Kundu et al. observed that blood group AB was most common among patients with severe PD [18]. Al-Askar’s systematic review showed that blood group O had the highest occurrence rate in PD, with a high event rate of positive Rh [19]. Table 1 summarizes studies related to ABO blood grouping in PD patients.

**Table 1:** Percentage of the ABO blood group in PD PD, periodontitis

Author	Year	A higher percentage of the ABO blood group in PD
Kaslick et al. [17]	1971	B +ve
Demir et al. [7]	2007	O +ve
Koregol et al. [12]	2010	O +ve
Vivek et al. [13]	2013	O +ve
Kundu et al. [18]	2014	AB +ve
Gautam et al. [15]	2017	B +ve
Al-Askar et al. [16]	2021	O +ve
Al-Askar [19]	2022	O +ve
Mostafa et al. [14]	2024	O +ve

Association of erythrocyte sedimentation rate (ESR) with the incidence of PD

The ESR is a commonly performed hematology test that may indicate and monitor an increase in inflammatory activity within the body caused by one or more conditions, such as autoimmune disease, infections, or tumors. The rate at which red blood cells settle in a tube is measured by the ESR test, indicating inflammation. Yadav et al. found a positive correlation between periodontal inflammation and ESR. They observed a statistically significant decrease during scaling and root planning in periodontal metrics and ESR [20]. Patel et al. found that nonsurgical periodontal care can reduce anemia in chronic PD patients and higher ESR and lower hemoglobin levels in periodontal disease patients, suggesting cytokines suppress erythropoiesis [21].

## Conclusions

The relationship between the ABO blood group and ESR in periodontal disorders remains disputed. Although studies suggest a potential relationship between the ABO blood group and periodontal health, the precise relationship between the ABO blood group and ESR in the context of periodontal disorders is unclear. Patients with blood type O show clinical symptoms of PD, but the high prevalence of the O blood group in the general population makes this finding unreliable. Larger sample sizes are needed to generate high-quality data for further research.
